# Safety, tolerability and pharmacokinetics of GSK3008348, a novel integrin αvβ6 inhibitor, in healthy participants

**DOI:** 10.1007/s00228-018-2435-3

**Published:** 2018-03-12

**Authors:** Charlotte H. Maden, David Fairman, Michelle Chalker, Maria J. Costa, William A. Fahy, Nadia Garman, Pauline T. Lukey, Tim Mant, Simon Parry, Juliet K. Simpson, Robert J. Slack, Stuart Kendrick, Richard P. Marshall

**Affiliations:** 1 Projects Clinical Platforms and Sciences, GSK, Uxbridge, Middlesex, UK; 2 Clinical Pharmacology Modelling and Simulation, GSK, Stevenage, Hertfordshire, UK; 3 Global Clinical Safety and Pharmacovigilance, GSK, Uxbridge, Middlesex, UK; 4 Clinical Statistics, GSK, Stevenage, Hertfordshire, UK; 5 Respiratory Therapy Area, GlaxoSmithKline, Gunnels Wood Road, Stevenage, Herts SG1 2NY UK; 6 QuintilesIMS, Reading, Berkshire, UK; 7 Platform Technology and Science, GSK, Ware, Hertfordshire, UK

**Keywords:** GSK3008348, Idiopathic pulmonary fibrosis, Pharmacokinetics, Safety, Integrin αvβ6 inhibitor

## Abstract

**Purpose:**

Inhaled drug delivery is an attractive route by which to deliver drugs to lungs of patients with idiopathic pulmonary fibrosis (IPF). GSK3008348 is a potent and selective small molecule being developed as the first inhaled inhibitor of the αvβ6 integrin for the treatment of IPF. The phase 1 first-time-in-human clinical trial (NCT02612051) presented here was designed to investigate the safety, tolerability and pharmacokinetic (PK) profile of single doses of GSK3008348 in healthy participants.

**Methods:**

Single ascending doses of GSK3008348 were administered to three cohorts of eight healthy participants in a randomised, double-blind, placebo-controlled, 4-period crossover design. Safety, tolerability and PK were assessed after single doses of 1–3000 mcg given by nebulisation.

**Results:**

A total of 29 participants were enrolled and received at least one dose of study treatment. There were no serious adverse events (AE) reported in any participant. No trends or clinically important differences were noted in the incidence or intensity of AEs or other safety assessments. Maximum plasma concentrations of GSK3008348 were generally attained within approximately 30 min after start of nebulisation, with geometric mean terminal elimination half-lives ranging from 7.95 to 10.2 h. Exposures, as measured by area under the plasma concentration-time curve (AUC), were dose proportional across all doses where estimates were possible (100–3000 mcg). Dose normalised geometric mean C_max_ increased with dose up to 3000 mcg. This supra proportionality was relatively modest, with a less than 3-fold increase over the range from 30 to 3000 mcg. The reason(s) for this observation are currently not known but may be due to slower absorption at the lowest doses. All exposures were within the exposure margins set by the non-clinical toxicity studies and so this is not expected to have any impact on safety.

**Conclusions:**

In summary, GSK3008348 was well tolerated at single doses up to 3000 mcg in healthy participants, and its PK profile was dose proportional at potentially clinically relevant doses (300–3000 mcg). These findings support further development of GSK3008348 as a novel inhaled treatment option for IPF.

**Electronic supplementary material:**

The online version of this article (10.1007/s00228-018-2435-3) contains supplementary material, which is available to authorized users.

## Introduction

Idiopathic pulmonary fibrosis (IPF) is a form of chronic, progressive fibrosing interstitial pneumonia with unknown aetiology, associated with a histological appearance of usual interstitial pneumonia. IPF progresses in a relentless and often insidious manner and is lethal, with median survival ranging from 2 to 4 years and a 5-year survival range of 20–30% [1, 2]. There are two disease-modifying treatments currently available to patients: pirfenidone and nintedanib. Both are oral anti-fibrotic therapies that demonstrate a reduction in the rate of decline in lung function (forced vital capacity, FVC) over 1 year [3, 4]. Pirfenidone is associated with phototoxicity, and both pirfenidone and nintedanib cause significant gastrointestinal effects, resulting in discontinuation due to adverse events (AEs) in approximately 30% of patients treated with pirfenidone and 20% of patients treated with nintedanib [5].

Development of new treatments for IPF has focussed on targets that drive fibrogenesis. It is likely that for maximal efficacy, a new medicine must reach the distal areas of the lung where fibrosis occurs. Aerosol-driven direct lung exposure combined with systemic drug availability may maximise the potential of a drug to reach all areas of the lung over a sustained period at lower doses than other routes of administration. GSK3008348 is a small molecule inhibitor of alpha-v beta-6 (αvβ6) integrin and represents the first inhaled compound in this class. The αvβ6 integrin plays a key role in the activation of transforming growth factor-β (TGFβ) [6] that is hypothesised to be central in the development of IPF [7, 8]. Expression of αvβ6 is significantly upregulated in IPF lung tissue [9] and localised to damaged epithelial sites [10]. Levels are also prognostic, with increased levels of αvβ6 observed in patients with rapidly progressing IPF [11]. Therefore, the αvβ6 integrin is an attractive therapeutic target for IPF with the advantage of inhibiting disease-specific activation of TGFβ via αvβ6, while limiting the potential for toxicological effects that may be associated with direct inhibition of TGFβ, that includes heart valve lesions and physeal dysplasia [12].

GSK3008348 demonstrates selectivity for αvβ6 and binds to the integrin with high affinity [13]. It has been shown to inhibit the activation of TGFβ with a prolonged duration of action in non-clinical in vitro and in vivo studies [14]. On delivery by nebulisation directly to the lungs, GSK3008348 is anticipated to bind to αvβ6 on the damaged epithelium and reduce TGFβ activation, thereby limiting the ability of TGFβ to promote collagen production and halting or slowing the progression of fibrosis. The study presented here was designed to investigate the safety, tolerability and pharmacokinetic (PK) profile of single doses of GSK3008348 in healthy participants.

## Methods

### Study design

This was a first-time-in-human, randomised, double-blind, single ascending-dose study of nebulised GSK3008348 (NCT02612051). GSK3008348 was formulated as a solution, administered via nebulisation over approximately 10 min using a Philips Respironics Inno Spire Delux Nebuliser with a Philips side stream mouthpiece. Informed consent was obtained from all individual participants in the study.

**Fig. 1:**
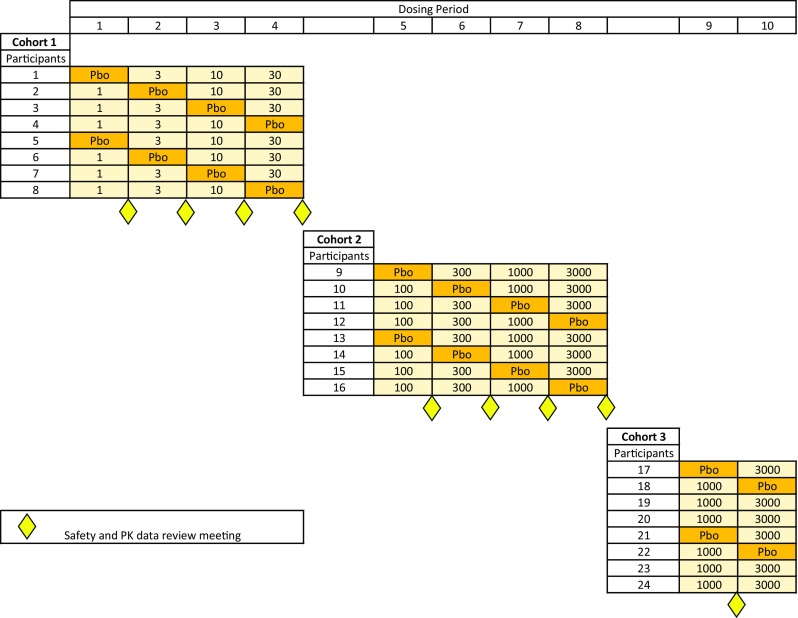
Study Schematic showing cohorts and doses in mcg. Participant numbers for illustrative purposes only, 5 additional subjects were recruited and dosed to replace participants that did not complete all dosing periods

Two cohorts of eight healthy participants each were randomised 3:1 to receive GSK3008348 (*n* = 6) or placebo (*n* = 2) in each period of a 4-period crossover. Within each cohort, participants were randomised to receive up to three ascending doses of GSK3008348 and one of placebo. In case it was necessary to repeat any doses to obtain more PK or safety data, a third cohort with the same design was also included in the study. Sentinel dosing was used throughout: on day 1, two subjects were dosed one active and one placebo, the remaining subjects in the dosing group were dosed the following day. Once each dose group had been completed, there was a blinded review of the safety and PK before proceeding to the next dose (Fig. 1).

In each period, participants were admitted to the clinical unit before dosing and were discharged 48 h after their dose. There was a washout period of at least 6 days between doses. This was based on non-clinical PK observations predicting an effective half-life of less than 12 h and human in vitro studies indicating a modestly extended pharmacodynamic effect. GSK3008348 demonstrates a receptor dissociation half-life of ~ 9 h and induces rapid internalisation of αvβ6 (minutes) followed by a slow return of the integrin to the cell surface (hours) due to integrin degradation [13, 14]. Non-clinical PK studies demonstrated rapid absorption of the drug from the lung into the systemic circulation with no evidence of significant lung retention. After the final dose, there was a follow-up period for 7–14 days.

Cohort 1 received 1, 3, 10, and 30 mcg GSK3008348, and cohort 2 received 100, 300, 1000, and 3000 mcg GSK3008348. Cohort 3 received a repeat of the highest dose levels, 1000 and 3000 mcg GSK3008348, to obtain additional information on safety and PK. The 1 mcg starting dose of GSK3008348 was predicted to engage approximately 15% of the αvβ6 in healthy lungs at lung peak concentrations, and the free systemic concentrations were predicted to be below the K_D_ at maximum plasma concentration (C_max_). This was a deliberately conservative approach given the novel mechanism. Dose escalation was continued, using half log steps, to a maximum of 3000 mcg in order to explore sustained higher occupancies (predicted > 90%) where downstream biomarker modulation and efficacy may be expected based on pre-clinical evidence [14]. In addition, 3000 mcg was the maximum dose supported by non-clinical toxicological studies. No systemic toxicity was identified at all doses tested in the 4-week inhalation toxicity studies. The human systemic exposures achieved across the dose range were within the systemic nonclinical safety margins.

The objectives of the study were achieved after the first two dosing periods in cohort 3; therefore, the study was terminated and the final two dosing periods in cohort 3 were not required.

### Study participants

Healthy male or female participants aged ≥ 18 years, with body weight ≥ 50 kg and body mass index ≥ 19.0–≤ 35.0 kg/m^2^, were eligible to participate in the study. Female participants were eligible if they were of non-childbearing potential. Participants were determined as healthy based on medical history, physical examination, clinical laboratory tests (haematology, clinical chemistry, urinalysis, HIV and hepatitis B and C screen, and drug, alcohol and smoking screening tests), 12-lead electrocardiogram (ECG), continuous cardiac telemetry, vital signs and pulmonary function tests.

Current smokers and participants with current or history of liver disease, known hepatic or biliary abnormalities, current or history of photosensitivity, current respiratory tract infection or history of excessive alcohol consumption were excluded.

### Safety assessments

Participants were under continuous medical supervision during each admission period (up to 48 h post-dose), and safety assessments were made at regular intervals throughout each period and until the end of follow-up. Assessment of safety was conducted by monitoring AEs, clinical laboratory tests (haematology, clinical chemistry and urinalysis), vital signs (including pulse oximetry), 12-lead ECGs, telemetry, physical examinations and pulmonary function tests (forced expiratory volume in 1 s [FEV_1_], FVC, diffusing capacity of the lung for carbon monoxide).

### PK assessments

Blood samples for the measurement of plasma GSK3008348 concentrations were taken up to 24 h after each dose administration and up to 30 h after dose administration in cohort 3. Analysis was conducted using a validated analytical method based on protein precipitation, followed by high-performance liquid chromatography (Waters Acquity Chromatograpy System) and mass spectrometry detection via an electrospray interface with multiple reaction monitoring (Waters Xevo TQ-S). The lower limit of quantification for GSK3008348 was 50 pg/mL using a 50-μL aliquot of human plasma with a higher limit of quantification of 50,000 pg/mL. The overall assay accuracy (% deviation from nominal) was ± 3.5% and precision (% coefficient of variation) was ≤ 8.4%.

### Statistical methods

Safety data was summarised descriptively and presented in tabular format. PK parameters were determined by non-compartmental analysis of GSK3008348 plasma concentration-time data. The following parameters were generated where data permitted: C_max_, time of maximum concentration (T_max_), terminal half-life (T_½_), area under the plasma concentration-time curve from zero hours to time (AUC_0-t_) and area under the plasma concentration-time curve from 0 h to infinity (AUC_0-inf_).

An assessment of dose proportionality was made for C_max_ and AUC_0-inf_ using the power model [15]. Both parameters were log_e_-transformed, and a mixed-effect model was fitted for each parameter with log_e_ (dose) as a fixed effect and subject specific intercepts as a random effect. For each parameter, the exponent of the power model and corresponding 90% confidence intervals (CIs) were estimated. Further dose proportionality analyses were performed using the analysis of variance method. Dose-normalised, log_e_-transformed AUC_0-inf_ and C_max_ were analysed separately using a mixed-effect model as described above but with dose as a categorical variable. Pairwise comparisons were made with respect to each dose versus the chosen reference dose of 300 mcg.

## Results

### Participant demographics and disposition

A total of 64 participants were screened, and 29 participants were enrolled and received at least one dose of GSK3008348 or placebo. Six participants were withdrawn: two due to AEs, three due to abnormal clinical laboratory safety tests and one due to a positive drug/alcohol screen on readmission. None of the AEs or abnormal clinical laboratory safety tests that led to withdrawal were attributed to GSK3008348. The demographics of the participants are summarised in Table 1.

**Table 1:** Summary of participant demographics

Demographics	Total *N* = 29
Age, years, mean (SD)	37.9 (10.2)
Sex, male, *n* (%)	29 (100)
Body mass index (kg/m^2^), mean (SD)	26.3 (2.2)
Height, cm, mean (SD)	175.4 (6.0)
Weight, kg, mean (SD)	80.9 (8.0)
Ethnicity, not Hispanic or Latino, *n* (%)	29 (100)
Race, *n* (%)	
African American/African heritage	2 (7)
Asian–Central/South Asian heritage	2 (7)
Asian–East Asian heritage	1 (3)
White	24 (83)

*SD* standard deviation

### Safety results

GSK3008348 was well tolerated at all dose levels, and there were no noted trends or clinically important differences in the incidence of AEs with escalating doses of GSK3008348 that would be suggestive of toxicity in humans. In fact, no treatment-related AEs were reported at the highest doses of 1000 or 3000 mcg GSK3008348. No serious AEs were reported in the study, and no participant was withdrawn due to a treatment-related AE.

Overall, 11 out of 29 participants experienced at least one AE in the study, all of which were considered mild in intensity. The two AEs that led to withdrawal of participants were a common cold, reported after a 3-mcg dose, and papular rash over the body, reported after a 100-mcg dose. Neither AE were considered to be related to study treatment. All AEs were resolved by follow-up, with the exception of the papular rash, which was ongoing at follow-up but was considered mild and unrelated to study treatment.

Five participants reported AEs that were considered related to study treatment, as summarised in Table 2; note that relatedness was assessed before unblinding of study treatment; therefore, some AEs were considered related to treatment with placebo. The most common treatment-related AEs were cough and dizziness, reported in two participants each.

**Table 2:** Summary of all participants who reported treatment-related AEs

Preferred term	Number (%) of participants									
	Placebo^a^	Dose level of GSK3008348, mcg								Total
		1	3	10	30	100	300	1000	3000	
	*N* = 20	*N* = 6	*N* = 6	*N* = 6	*N* = 6	*N* = 6	*N* = 6	*N* = 12	*N* = 11	*N* = 29
Any AE	2 (10)	2 (33)	0	2 (33)	0	1 (17)	1 (17)	0	0	5 (17)
Cough	1 (5)	1 (17)	0	1 (17)	0	1 (17)	0	0	0	2 (7)
Dizziness	1 (5)	0	0	1 (17)	0	0	0	0	0	2 (7)
Headache	0	1 (17)	0	0	0	0	0	0	0	1 (3)
Lethargy	0	0	0	1 (17)	0	0	0	0	0	1 (3)
Rhinorrhoea	0	0	0	0	0	0	1 (17)	0	0	1 (3)

*AEs* adverse events

^a^Relatedness to treatment was recorded before study unblinding

Note: table shows number of participants who reported each AE; participants could have reported more than one AE

There were no clinically significant abnormal clinical laboratory values. All 12-lead ECG, telemetry and vital sign measurements outside normal ranges were deemed not clinically significant. Analysis of individual change from baseline in FEV_1_ and FVC over time did not show clinically significant differences between the treatment groups (Supplementary Table 1). No notable trends were observed in clinical laboratory parameters, ECGs, vital signs and pulmonary function test values during the course of the study or between treatment groups.

### PK results

**Fig. 2:**
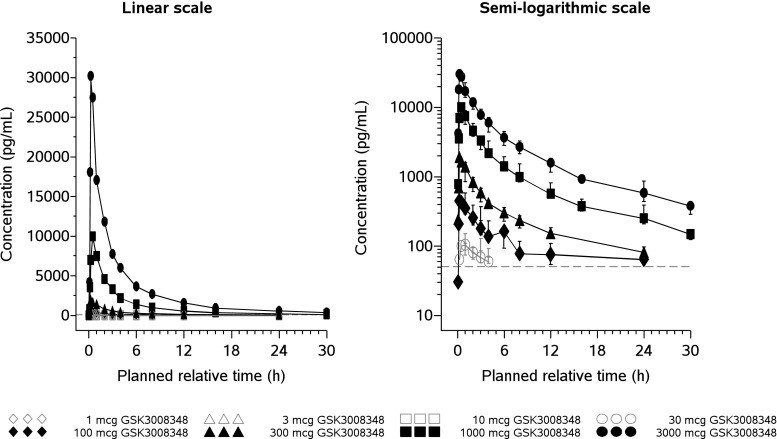
Median plasma GSK3008348 concentration-time plots by treatment with single nebulised doses of 1–3000 mcg GSK3008348, shown on linear and semi-logarithmic scales. For the semi-logarithmic scale plot, error bars are 25 to 75% quartiles

Median plasma concentrations at each nominal time point for each treatment are summarised in Fig. 2. At the lowest dose levels of 1, 3 and 10 mcg GSK3008348, plasma concentrations were not quantifiable. Plasma concentrations of GSK3008348 were measurable between 0.25 and 4 h post-dose in all but one participant following 30-mcg doses, and up to 24 h post-dose following doses of 100 mcg and higher. Plasma concentrations were also measurable up to 30 h post-dose following 1000- and 3000-mcg doses. The plasma concentration-time profiles showed rapid absorption of GSK3008348 up to 1–3 h post-dose, followed by rapid decline 4–12 h post-dose, followed by a slower decline with geometric mean T_½_ ranging from 7.95 to 10.2 h, suggesting multi-phasic elimination of GSK3008348. A summary of derived GSK3008348 PK parameters following single nebulised doses of 1–3000 mcg GSK3008348 are presented in Table 3 and selected dose normalised parameters in Table 4. Lower variability in the GSK3008348 plasma concentration profiles was observed between participants at doses of 300, 1000 and 3000 mcg compared with the lower doses (Table 3).

**Table 3:** Summary of derived PK parameters following single nebulised doses of 1–3000 mcg GSK3008348

PK parameter (units)	Cohort	GSK3008348 treatment regimen, mcg	*N*	*n*	n*	Geometric mean (%CVb)	95% CI
AUC_(0-t)_ (h*pg/mL)	1^a^	30	6	6	1	285 (82.1)	(134, 605)
	2^a^	100	6	6	0	1765 (99.0)	(741, 4203)
		300	6	6	0	6850 (25.7)	(5252, 8934)
		1000	6	6	0	29,093 (45.3)	(18,494, 45,766)
		3000	5	5	0	82,205 (27.9)	(58,519, 115,476)
	3^b^	1000	6	6	0	38,512 (26.8)	(29,201, 50,791)
		3000	6	6	0	93,360 (20.1)	(75,777, 115,023)
AUC_(0-inf)_ (h*pg/mL)	1	30	6	1	1	NE	NE
	2	100	6	4	0	3527 (52.5)	(1610, 7729)
		300	6	6	0	8041 (24.7)	(6226, 10,384)
		1000	6	6	0	32,758 (46.1)	(20,666, 51,925)
		3000	5	5	0	89,605 (29.7)	(62,463, 128,540)
	3	1000	6	6	0	40,773 (27.2)	(30,795, 53,982)
		3000	6	6	0	98,343 (19.8)	(80,047, 120,820)
C_max_ (pg/mL)	1	30	6	6	1	102 (106)	(41.2, 254)
	2	100	6	6	0	501 (46.8)	(314, 798)
		300	6	6	0	1922 (42.1)	(1258, 2938)
		1000	6	6	0	7243 (42.5)	(4723, 11,108)
		3000	5	5	0	29,393 (24.6)	(21,749, 39,724)
	3	1000	6	6	0	12,596 (16.6)	(10,593, 14,979)
		3000	6	6	0	30,443 (16.9)	(25,535, 36,294)
T_max_^c^ (h)	1	30	6	5	0	1.02 (0.52,3.02)	
	2	100	6	4	0	0.517 (0.27,1.02)	
		300	6	6	0	0.392 (0.27,1.02)	
		1000	6	6	0	0.392 (0.27,1.02)	
		3000	5	5	0	0.267 (0.27,0.52)	
	3	1000	6	6	0	0.517 (0.27,0.52)	
		3000	6	6	0	0.267 (0.27,0.52)	
T_½_ (h)	1	30	6	0	0	NE	NE
	2	100	6	4	0	10.2 (27.8)	(6.62, 15.8)
		300	6	6	0	10.1 (19.3)	(8.23, 12.3)
		1000	6	6	0	8.97 (20.2)	(7.27, 11.1)
		3000	5	5	0	7.95 (8.0)	(7.20, 8.78)
	3	1000	6	6	0	9.45 (16.2)	(7.98, 11.2)
		3000	6	6	0	8.93 (14.6)	(7.67, 10.4)

Note: all GSK3008348 plasma concentrations were non-quantifiable following administration of 1, 3, and 10 mcg GSK3008348

*AUC*_*0-t*_ area under the plasma concentration-time curve from zero hours to time, *AUC*_*0-inf*_ area under the plasma concentration-time curve from zero hours to infinity, *CI* confidence interval, *C*_*max*_ maximum plasma concentration, *%CVb* between-participant coefficient of variation, *NE* not evaluable, *PK* pharmacokinetic, *T*_*½*_ terminal half-life, *T*_*max*_ time of maximum concentration

^a^Sampling up to 24 h post-dose

^b^Sampling up to 30 h post-dose

^c^T_max_ expressed as median (range)

*N* number of participants dosed with active treatment in each cohort, *n* number of participants with non-missing observations (including imputed values that were non-calculable due to non-quantifiable concentrations), n* number of subjects for whom parameter could not be derived because of non-quantifiable concentrations

**Table 4:** Dose normalised geometric mean GSK3008348 PK parameters

PK parameter (units)	Cohort	GSK3008348 treatment regimen, mcg	*N*	*n*	n*	Geometric mean (%CVb)	95% CI
AUC_(0-inf)_/D (h*pg/mL)/mcg	1	30	6	1	1	NE	NE
	2	100	6	4	0	35.3 (52.5)	(16.0, 77.3)
		300	6	6	0	26.8 (24.7)	(20.8, 34.6)
		1000	6	6	0	32.8 (46.1)	(20.7, 51.9)
		3000	5	5	0	29.9 (29.7)	(20.8, 42.9)
	3	1000	6	6	0	40.8 (27.2)	(30.8, 54.0)
		3000	6	6	0	32.8 (19.8)	(26.7, 40.3)
AUC_(0-t)_/D (h*pg/mL)/mcg	1	30	6	6	1	9.05 (96.1)	(3.9, 21.2)
	2	100	6	6	0	17.7 (99.0)	(7.4, 42.0)
		300	6	6	0	22.8 (25.7)	(17.5, 29.8)
		1000	6	6	0	29.1 (45.3)	(18.5, 45.8)
		3000	5	5	0	27.4 (27.9)	(19.5, 38.5)
	3	1000	6	6	0	38.5 (26.8)	(29.2, 50.8)
		3000	6	6	0	31.1 (20.1)	(25.3, 38.3)
C_max_/D (pg/mL)/mcg	1	30	6	6	1	3.41 (106.0)	(1.4, 8.5)
	2	100	6	6	0	5.01 (46.8)	(3.1, 8.0)
		300	6	6	0	6.41 (42.1)	(4.2, 9.8)
		1000	6	6	0	7.24 (42.5)	(4.7, 11.1)
		3000	5	5	0	9.80 (24.6)	(7.3, 13.2)
	3	1000	6	6	0	12.6 (16.6)	(10.6, 15.0)
		3000	6	6	0	10.2 (16.9)	(8.5, 12.1)

*AUC*_*0-inf*_ area under the plasma concentration-time curve from 0 h to infinity, *CI* confidence interval, *C*_*max*_ maximum plasma concentration, *%CVb* between-participant coefficient of variation, *NE* not evaluable

Systemic exposure of GSK3008348, as measured by geometric mean AUC_0-inf_, AUC_0-t_ and C_max_, increased with escalating dose. There appeared to be no trend in the dose normalised geometric mean AUC_0-inf_ estimates, with the lowest dose normalised geometric mean observed at 300 mcg (26.8 pg*h/mL) and the highest adjusted geometric mean observed at 100 mcg (35.3 pg*h/mL). In contrast, dose normalised geometric means for C_max_ increased with escalating doses of GSK3008348, although they appeared to plateau above 1000 mcg (Table 4). This effect may have been due to moderately slower absorption from the lung at lower doses, as the T_max_ was latest at the 30-mcg dose and a later upper range was observed at doses lower than 3000 mcg (Table 3).

Inter-individual variability in the extent of systemic exposure to GSK3008348 ranged from low to high, with geometric coefficient of variance (CV) for C_max_, AUC_0-t_ and AUC_0-inf_ ranging from 16.6 to 106%, 20.1 to 99.0% and 19.8 to 52.5%, respectively, across all dose groups where quantifiable plasma concentrations were observed.

### Dose proportionality assessment

Dose normalised AUC_(0-inf)_ and C_max_ are summarised in Table 4 and show that AUC_(0-inf)_ generally increased in a dose proportional manner. Consistent with that observation, a preliminary assessment of dose proportionality between subjects was made using the power model with the exponent of the power model fitted to AUC_(0-inf)_ estimated at 0.98 (90% CI: 0.90, 1.05) (Supplementary Table 2).

There was a clear trend for the dose normalised geometric mean C_max_ to increase with dose up to 3000 mcg. The increase was relatively modest, with a less than 3-fold increase over the range from 30 to 3000 mcg. The exponent of the power model fitted to C_max_ was estimated at 1.21 (90% CI: 1.13, 1.29) (Supplementary Table 2). At the 1000- and 3000-mcg doses, dose normalised geometric means remained constant, suggesting that dose proportionality for C_max_ was achieved at higher doses (Supplementary Table 3; Supplementary Fig. 1).

## Discussion

Treatment of IPF remains a significant unmet need, despite the recent availability of two licenced medicines: pirfenidone and nintedanib. Current treatments reduce the rate of decline in lung function, but do not stop progression of IPF altogether. Blockade of αvβ6 integrin represents an attractive anti-fibrotic drug mechanism, with a body of evidence to suggest that it plays a central role in the activation of TGFβ and the pathogenesis of lung fibrosis [16]. By virtue of its restricted expression, αvβ6 represents a more targeted approach to modulating the process of fibrogenesis that results in the laying down of fibrotic tissue in the lung. This has the potential to confer advantages compared with approaches that seek to achieve a more generalised blockade of TGFβ, which has been shown to have negative side effects given TGFβ’s role in a variety of normal physiological processes [17]. Furthermore, in vitro experiments suggest that pirfenidone and nintedanib have limited effects on αvβ6 integrin-mediated TGFβ activation, indicating that targeting this pathway has the potential to provide mechanistic differentiation [18]. These observations also suggest that this approach could be attractive as an add-on therapy to either pirfenidone or nintedanib, as part of a multi-faceted approach to arrest fibrosis, the importance of which has been extensively discussed [19].

GSK3008348 is the first compound in the αvβ6 integrin class to be administered via the inhaled route. In addition to αvβ6 integrin being an attractive mechanism to target localised TGFβ activation in areas of fibrotic lung, delivery of GSK3008348 via inhalation may confer additional advantages compared with oral and intravenous routes of administration. Aerosol-driven direct lung exposure combined with systemic drug availability may maximise the potential of a drug to reach all areas of the lung over a sustained period at lower doses than other routes of administration.

The results from this first-time-in-human, single ascending-dose study provide support for the continued development of GSK3008348 as an inhaled therapy for the treatment of IPF. Safety data collected in the study suggest that GSK3008348 was well tolerated in healthy participants at doses up to 3000 mcg. Importantly, inhalation of GSK3008348 was not associated with local or systemic adverse effects, demonstrating that the inhaled route is a viable one that can be used in future patient studies.

The PK profile for single doses of GSK3008348 was successfully characterised, indicating maximal concentrations at approximately 30 min post-start of nebulisation and geometric mean terminal T_½_ ranging from 7.95 to 10.2 h. Increases in systemic exposure, defined by the area under the concentration curve, were dose proportional. There was a clear trend for the dose normalised geometric mean C_max_ to increase with doses up to 3000 mcg. The increase was relatively modest, with a less than 3-fold increase over the range from 30 to 3000 mcg and may have been due to a slower absorption from the lung at the lowest doses. The implication of the observed PK profile suggests that a twice daily dosing regimen may be efficacious (assuming that lung exposures parallel those in the systemic circulation). An investigational study to determine levels of target engagement in the lung is ongoing and will be utilised to further refine potential dosing regimens (NCT03069989).

It is expected, based on recent comparative observations of the PK of nebulised salbutamol in healthy participants and patients with IPF (unpublished data), that nebulised GSK3008348 would have a similar PK profile in patients with IPF to that observed in the healthy participants in the current study.

Results from this study therefore support the progression of GSK3008348 to the next stage of clinical development. Establishing the safety profile of repeated doses is a key next step, as is investigating the effects of single doses of GSK3008348 on target engagement in patients with IPF (NCT03069989).

## Conclusion

This study has demonstrated that GSK3008348, an αvβ6 integrin inhibitor administered as an inhaled solution, was well tolerated, and systemic exposure was linear at doses of 300–3000 mcg. Taken together with results from preclinical studies, these findings support further development of inhaled GSK3008348 for the treatment of IPF.

## Electronic supplementary material

ESM 1 (DOCX 171 kb)
