# Supplementary material for: Safety, tolerability and pharmacokinetics of GSK3008348, a novel integrin αvβ6 inhibitor, in healthy participants
Source: Eur J Clin Pharmacol. 2018 Mar 12;74(6):701–9. doi: 10.1007/s00228-018-2435-3 (PMC5942357; doi:10.1007/s00228-018-2435-3)
Supplement: Supplementary file 1 — (DOCX 171 kb) [file 228_2018_2435_MOESM1_ESM.docx]

Safety, tolerability and pharmacokinetics of GSK3008348, a novel integrin αvβ6 inhibitor, in healthy participants

Charlotte H Maden,^1^ David Fairman,^2^ Michelle Chalker,^3^ Maria J Costa,^4^ William A Fahy,^5^ Nadia Garman,^1^ Pauline T Lukey,^5^ Tim Mant,^6^ Simon Parry,^7^ Juliet K Simpson,^5^ Robert J Slack^5^, Stuart Kendrick,^5^ Richard P Marshall^5^

^1^Projects Clinical Platforms & Sciences, GSK, Uxbridge, Middlesex, UK; ^2^Clinical Pharmacology Modelling and Simulation, GSK, Stevenage, Hertfordshire, UK; ^3^Global Clinical Safety & Pharmacovigilance, GSK, Uxbridge, Middlesex, UK; ^4^Clinical Statistics, GSK, Stevenage, Hertfordshire, UK; ^5^Respiratory Therapy Area, GSK, Stevenage, Hertfordshire, UK; ^6^QuintilesIMS, Reading, Berkshire, UK; ^7^Platform Technology & Science, GSK, Ware, Hertfordshire, UK.

**Email address of corresponding author:** [richard.p.marshall@gsk.com](mailto:richard.p.marshall@gsk.com)

### Journal: *European Journal of Clinical Pharmacology*

### Online resources

Supplementary Table 1: Summary of change from baseline to 24 h post dose of absolute lung function values (DLCO, FEV_1_ and FVC) by treatment group

|  |  |  |  | |  | |  | |  | |  | |  | |  | |  | |
| --- | --- | --- | --- | --- | --- | --- | --- | --- | --- | --- | --- | --- | --- | --- | --- | --- | --- | --- |
|  |  | Placebo | | 1 mcg | | 3 mcg | | 10 mcg | | 30 mcg | | 100 mcg | | 300 mcg | | 1000 mcg | | 3000 mcg |
|  |  | (N=20) | | (N=6) | | (N=6) | | (N=6) | | (N=6) | | (N=6) | | (N=6) | | (N=12) | | (N=11) |
| DLCO (L) | Mean | 0.234 | | -0.153 | | 0.938 | | 0.113 | | -0.168 | | -0.862 | | 0.562 | | -0.293 | | 0.547 |
|  | SD | 0.8669 | | 0.9344 | | 0.8022 | | 0.784 | | 0.9226 | | 0.8313 | | 0.607 | | 0.3144 | | 0.484 |
|  |  | | | | | | | | | | | | | | | | | |
| FEV_1_ (L) | Mean | -0.005 | | -0.043 | | -0.007 | | -0.053 | | 0.085 | | 0.023 | | -0.072 | | -0.027 | | -0.025 |
|  | SD | 0.0738 | | 0.0891 | | 0.1392 | | 0.1009 | | 0.094 | | 0.1211 | | 0.1264 | | 0.0853 | | 0.1588 |
|  |  | | | | | | | | | | | | | | | | | |
| FVC (L) | Mean | 0.006 | | -0.06 | | 0.125 | | -0.105 | | 0.033 | | 0.12 | | -0.078 | | -0.014 | | -0.094 |
|  | SD | 0.1244 | | 0.0844 | | 0.309 | | 0.1263 | | 0.0344 | | 0.0684 | | 0.0773 | | 0.1212 | | 0.1558 |

N = Number of participants dosed.

DLCO, diffusing capacity of the lung for carbon monoxide; FEV_1_, forced expiratory volume in 1 second; FVC, forced vital capacity; SD, standard deviation.

Supplementary Table 2: Summary of statistical analysis on dose proportionality of plasma GSK3008348 PK parameters (power model)

| **PK parameter (units)** | **Exponent of the power model** | |
| --- | --- | --- |
|  | **Estimate** | **90% CI** |
| AUC_(0-inf)_ (h*pg/mL) | 0.98 | (0.90, 1.05) |
| C_max_ (pg/mL) | 1.21 | (1.13, 1.29) |

AUC_0-inf_, area under the plasma concentration-time curve from zero hours to infinity; CI, confidence interval; C_max_, maximum plasma concentration; PK, pharmacokinetic.

The exponent of the power model fitted to AUC_0-inf_ and C_max_ were estimated at 0.98 (90% CI: 0.90, 1.05) and 1.21 (90% CI: 1.13, 1.29), respectively. The exponent for AUC_(0-inf)_ is close to 1, and the 90% CI includes 1, which can be interpreted as a dose proportional relationship. However, the lower bound of the 90% CI for C_max_ is greater than 1 and thus over the wide dose range of 30–3000 mcg, there is a tendency for C_max_ to increase in a greater than dose proportional relationship. This was further confirmed with additional exploratory analysis using the ANOVA model, the results of which are displayed in Supplementary Table 3. There is a clear trend for the dose normalised geometric mean C_max_ to increase with dose up to 1000 mcg. However, at the higher doses of 1000 and 3000 mcg, dose normalised geometric means remained constant, suggesting dose proportionality for C_max_ could be achieved at higher doses.

Adjusted geometric mean ratios of the dose normalised PK parameters relative to the reference dose of 300 mcg, with 90% CIs from the ANOVA analysis are presented in Supplementary Table 3.

Supplementary Table 3: Summary of ratio of dose normalised AUC_(0-inf)_ and C_max_ relative to the 300-mcg dose (ANOVA)

| **GSK3008348**  **Test treatment** | **Ratio (test / 300 mcg) of geometric means (90% CI)** | |
| --- | --- | --- |
|  | **AUC_(0-inf)_/D** | **C_max_/D** |
| 30 mcg | NA | 0.44 (0.28, 0.70) |
| 100 mcg | NA | 0.73 (0.51, 1.05) |
| 1000 mcg | 1.16 (1.00,1.36) | 1.24 (0.89, 1.72) |
| 3000 mcg | 0.98 (0.84, 1.14)^a^ | 1.25 (0.89, 1.74) |
| AUC_0-inf_, area under the plasma concentration-time curve from zero hours to infinity; CI, confidence interval; C_max_, maximum plasma concentration; NA, not applicable. Analysis of AUC_(0-inf)_ was based on dose groups at 300 mcg and higher.  ^a^90% CI falls entirely within standard equivalence criteria (0.80, 1.25) demonstrating dose proportionality. | | |

The greater than dose proportional relationship in C_max_ was particularly evident when 30 mcg is compared with 300 mcg with an estimated ratio of 0.44 and the upper bound of the 90% CI being below 1. All other 90% CIs included 1, thus there is no statistically significant deviation from dose proportionality at doses above 300 mcg at the 10% significance level.

**Supplementary Figure 1. Treatment ratios in (a) AUC_(0-inf);_ GSK3008348 300 mcg versus 1000 and 3000 mcg and (b) C_max_; GSK3008348 300 mcg versus 30, 100, 1000 and 3000 mcg**
